# 

**Published:** 1887-01

**Authors:** 


PAGE.

ORIGINAL COMMUNICATIONS—Tape Worms in Birds. Prof. Joseph Leidy, M. D., LL. D. ...... 1
A Comparative Study of Sebaceous Cysts and Cutaneous Horns. John Bland Sutton, F. R. C. S. 11
A Contribution to the Study of the Microbe of Rabbit Septicæma. Theobald Smith, M. D......... 24
Snake Poison from a Chemico-Physiological Point of View. T. Wesley Mills, M. A., M. D., L. R. C. P. ........ 38
Comparative Ophthalmology. Clinical Observations. L. Webster Fox, M. D........ 43
Breeding and Feeding. A. S. Heath, M. D........ 47
Lung Worms in Sheep. N. S. Townsend, M. D........ 56
"Barsati," or Atrophic Carcinoma. Richard W. Burke, V. S., A. D. V........ 60
EDITORIAL DEPARTMENT........ 66
CASE DEPARTMENT........ 71
SELECTIONS FROM FOREIGN PERIODICALS........ 78
PROCEEDINGS VETERINARY MEDICAL SOCIETIES........ 84
PROGRESS OF VETERINARY SCIENCE........ 95
REVIEWS........ 99

